# Supplementary material for: Discovery and application of insertion-deletion (INDEL) polymorphisms for QTL mapping of early life-history traits in Atlantic salmon
Source: BMC Genomics. 2010 Mar 8;11:156. doi: 10.1186/1471-2164-11-156 (PMC2838853; doi:10.1186/1471-2164-11-156)
Supplement: Additional file 2 — Information on developed 76 locus single-run INDEL panel in Atlantic salmon. Information on fluorescence labeling, primer concentrations, PCR pooling and links to alignments, INDEL motifs and GENESCAN (Burge and Karlin 1997) predictions of genes/exons are available in html format. [file 1471-2164-11-156-S2.ZIP › Additionalfile2/snpsummary4707.html]

```
Cluster 1123 Contig 1

prev  Summary    Contig List  next
```

Size of Consensus sequence = 1207

Number of sequences = 33

Minimum redundancy = 6

Key

A gi|117516621|gb|EG848380.1|EG848380 EST\_ssal\_eve\_5934 ssaleve thyroid Salmo salar cDNA Salmo salar cDNA clone ssal\_eve\_507\_026\_fwd 3', mRNA sequence  
B gi|117861229|gb|EG933925.1|EG933925 EST\_ssal\_evf\_35691 ssalevf mixed\_tissue Salmo salar cDNA Salmo salar cDNA clone ssal\_evf\_547\_199\_fwd 3', mRNA sequence  
C gi|117865439|gb|EG938135.1|EG938135 EST\_ssal\_evf\_21890 ssalevf mixed\_tissue Salmo salar cDNA Salmo salar cDNA clone ssal\_evf\_527\_307\_fwd 3', mRNA sequence  
D gi|117832617|gb|EG905313.1|EG905313 EST\_ssal\_evf\_7938 ssalevf mixed\_tissue Salmo salar cDNA Salmo salar cDNA clone ssal\_evf\_508\_365\_fwd 3', mRNA sequence  
E gi|24354721|gb|CA048551.1|CA048551 ssalsrkh001002 kidney Salmo salar cDNA, mRNA sequence  
F gi|29327924|gb|CB516698.1|CB516698 ssalrgb555071 mixed\_tissue Salmo salar cDNA, mRNA sequence  
G gi|117513843|gb|EG845602.1|EG845602 EST\_ssal\_eve\_58333 ssaleve thyroid Salmo salar cDNA Salmo salar cDNA clone ssal\_eve\_579\_195\_fwd 3', mRNA sequence  
H gi|117513844|gb|EG845603.1|EG845603 EST\_ssal\_eve\_58334 ssaleve thyroid Salmo salar cDNA Salmo salar cDNA clone ssal\_eve\_579\_195\_rev 5', mRNA sequence  
I gi|117865438|gb|EG938134.1|EG938134 EST\_ssal\_evf\_21889 ssalevf mixed\_tissue Salmo salar cDNA Salmo salar cDNA clone ssal\_evf\_527\_307\_rev 5', mRNA sequence  
J gi|117516622|gb|EG848381.1|EG848381 EST\_ssal\_eve\_5935 ssaleve thyroid Salmo salar cDNA Salmo salar cDNA clone ssal\_eve\_507\_026\_rev 5', mRNA sequence  
K gi|117861230|gb|EG933926.1|EG933926 EST\_ssal\_evf\_35692 ssalevf mixed\_tissue Salmo salar cDNA Salmo salar cDNA clone ssal\_evf\_547\_199\_rev 5', mRNA sequence  
L gi|117501324|gb|EG833342.1|EG833342 EST\_ssal\_eve\_10749 ssaleve thyroid Salmo salar cDNA Salmo salar cDNA clone ssal\_eve\_514\_220\_fwd 3', mRNA sequence  
M gi|117854929|gb|EG927625.1|EG927625 EST\_ssal\_evf\_30019 ssalevf mixed\_tissue Salmo salar cDNA Salmo salar cDNA clone ssal\_evf\_539\_323\_fwd 3', mRNA sequence  
N gi|45313337|gb|CK883706.1|CK883706 SGP167623 Atlantic salmon Heart cDNA library Salmo salar cDNA clone H5-0853 5', mRNA sequence  
O gi|117869032|gb|EG941728.1|EG941728 EST\_ssal\_evf\_25124 ssalevf mixed\_tissue Salmo salar cDNA Salmo salar cDNA clone ssal\_evf\_533\_100\_rev 5', mRNA sequence  
P gi|117501335|gb|EG833353.1|EG833353 EST\_ssal\_eve\_10750 ssaleve thyroid Salmo salar cDNA Salmo salar cDNA clone ssal\_eve\_514\_220\_rev 5', mRNA sequence  
Q gi|24388754|gb|CA058511.1|CA058511 ssalrga506092 mixed\_tissue Salmo salar cDNA, mRNA sequence  
R gi|24382207|gb|CA051964.1|CA051964 ssalrgb510134 mixed\_tissue Salmo salar cDNA, mRNA sequence  
S gi|25998826|gb|CA769571.1|CA769571 ssalrgb522349 mixed\_tissue Salmo salar cDNA, mRNA sequence  
T gi|25999602|gb|CA770347.1|CA770347 ssalrga516350 mixed\_tissue Salmo salar cDNA, mRNA sequence  
U gi|85022194|gb|DW550850.1|DW550850 EST\_ssal\_rgb2\_15269 rgb2 Salmo salar cDNA clone ssal\_rgb2\_525\_126\_fwd 3', mRNA sequence  
V gi|85042577|gb|DW570755.1|DW570755 EST\_ssal\_rgb2\_35174 rgb2 Salmo salar cDNA clone ssal\_rgb2\_556\_345\_fwd 3', mRNA sequence  
W gi|89867612|gb|DY723735.1|DY723735 EST\_ssal\_rgb2\_79474 ssalrgb2 mixed\_tissue Salmo salar cDNA Salmo salar cDNA clone ssal\_rgb2\_630\_275\_fwd 3', mRNA sequence  
X gi|117535106|gb|EG866551.1|EG866551 EST\_ssal\_eve\_28088 ssaleve thyroid Salmo salar cDNA Salmo salar cDNA clone ssal\_eve\_538\_068\_rev 5', mRNA sequence  
Y gi|117832616|gb|EG905312.1|EG905312 EST\_ssal\_evf\_7937 ssalevf mixed\_tissue Salmo salar cDNA Salmo salar cDNA clone ssal\_evf\_508\_365\_rev 5', mRNA sequence  
Z gi|117569119|gb|EG890095.1|EG890095 EST\_ssal\_evf\_3610 ssalevf mixed\_tissue Salmo salar cDNA Salmo salar cDNA clone ssal\_evf\_503\_049\_rev 5', mRNA sequence  
a gi|117569130|gb|EG890106.1|EG890106 EST\_ssal\_evf\_3611 ssalevf mixed\_tissue Salmo salar cDNA Salmo salar cDNA clone ssal\_evf\_503\_049\_fwd 3', mRNA sequence  
b gi|117854930|gb|EG927626.1|EG927626 EST\_ssal\_evf\_30020 ssalevf mixed\_tissue Salmo salar cDNA Salmo salar cDNA clone ssal\_evf\_539\_323\_rev 5', mRNA sequence  
c gi|24354990|gb|CA048820.1|CA048820 ssalkhc004046 kidney Salmo salar cDNA, mRNA sequence  
d gi|85042156|gb|DW570334.1|DW570334 EST\_ssal\_rgb2\_34753 rgb2 Salmo salar cDNA clone ssal\_rgb2\_556\_097\_fwd 3', mRNA sequence  
e gi|117490175|gb|EG822392.1|EG822392 EST\_ssal\_evd\_24133 ssalevd thymus Salmo salar cDNA Salmo salar cDNA clone ssal\_evd\_531\_132\_fwd 3', mRNA sequence  
f gi|117490174|gb|EG822391.1|EG822391 EST\_ssal\_evd\_24132 ssalevd thymus Salmo salar cDNA Salmo salar cDNA clone ssal\_evd\_531\_132\_rev 5', mRNA sequence  
g gi|24335317|gb|CA036457.1|CA036457 ssalob001092 reproductive Salmo salar cDNA, mRNA sequence

2 SNPs detected

A B C D E F G H I J K L M N O P Q R S T U V W X Y Z a b c d e f g  cosegregation weighted

769 . . . - - - - - - - - - - - . - - T T T T - - - . - - - - T T T T   2/2 84.85
770 . . . - - - - - - - - - - - . - - T T T T - - - . - - - - T T T T   2/2 84.85
